# The Male Warrior Hypothesis: Testosterone-related Cooperation and Aggression in the Context of Intergroup Conflict

**DOI:** 10.1038/s41598-019-57259-0

**Published:** 2020-01-15

**Authors:** J. A. Muñoz-Reyes, P. Polo, N. Valenzuela, P. Pavez, O. Ramírez-Herrera, O. Figueroa, C. Rodriguez-Sickert, D. Díaz, M. Pita

**Affiliations:** 1 Laboratorio de Comportamiento Animal y Humano, Centro de Estudios Avanzados, Universidad de Playa Ancha, Valparaíso, Chile; 2 Departamento de Ciencias Biológicas, Facultad de Ciencias de la Vida, Universidad Andrés Bello, Viña del Mar, Chile; 3 Centro de Investigación en Complejidad Social, Facultad de Gobierno, Universidad del Desarrollo, Santiago, Chile; 4 Facultad de Ciencias Económicas, Universidad de Chile, Santiago, Chile; 5 Departamento de Biología, Universidad Autónoma de Madrid, Madrid, Spain

**Keywords:** Human behaviour, Sexual selection

## Abstract

The Male Warrior Hypothesis (MWH) establishes that men’s psychology has been shaped by inter-group competition to acquire and protect reproductive resources. In this context, sex-specific selective pressures would have favored cooperation with the members of one’s group in combination with hostility towards outsiders. We investigate the role of developmental testosterone, as measured indirectly through static markers of prenatal testosterone (2D:4D digit ratio) and pubertal testosterone (body musculature and facial masculinity), on both cooperation and aggressive behavior in the context of intergroup conflict among men. Supporting the MWH, our results show that the intergroup conflict scenario promotes cooperation within group members and aggression toward outgroup members. Regarding the hormonal underpinnings of this phenomenon, we find that body musculature is positively associated with aggression and cooperation, but only for cooperation when context (inter-group competition) is taken into account. Finally, we did not find evidence that the formidability of the group affected individual rates of aggression or cooperation, controlling for individual characteristics.

## Introduction

Human beings are adapted to living in social groups. This pattern of behavior has shaped the evolution of the human mind, favoring behavioral strategies that benefit group formation and cohesion through cooperation with non-genetically related individuals^1^. Living in groups provides enormous benefits in relation to hunting and protection against predators, but also creates scenarios of inter-group conflict. Moreover, it has been argued that intra-group cooperation co-evolved with hostility towards outsiders^2,3^.

Archaeological and comparative evidence indicates that inter-group conflict has been present since the dawn of our lineage^4^. Anthropological studies of tribal societies in the 20^th^ and 21^st^ centuries have shed light on the benefits of inter-group conflict for males^5^, shaped by sexual selection to compete through physical aggression^6,7^. The male warrior hypothesis argues that “humans, particularly men, may possess psychological mechanisms enabling them to form coalitions capable of planning, initiating and executing acts of aggression on members of outgroups, with the ultimate goal of acquiring or protecting reproductive resources”^8^. In this context, testosterone has been shown to play an important role in cooperative and aggressive behaviors, functioning as a status-seeking hormone^9^. However, little research has been done on the effect of this hormone on levels of aggression and cooperation in the context of intergroup conflict among men.

The male warrior hypothesis is a sex-specific proposal primarily supported theoretically by the greater degree of variance among men than among women in terms of reproductive success^10,11^ and the lower level of obligatory parental investment by men^12^. These two factors have enhanced intrasexual competition in men, thus favoring sexual dimorphism in size and strength, accompanied by a significant sex-based difference in physical aggressiveness^6^. In this sense, men have enormous incentives to form coalitions to be involved in intergroup contests because of the benefits associated with winning these contests, even if the costs of losing the contest could be devastating^13^. Therefore, in contrast to women, men can exacerbate intra-group cooperation^14^ and intergroup aggression^15^ in the context of an intergroup threat, the most important factor triggering these behaviors being the incentives to monopolize resources. Moreover, exacerbation of intergroup conflict in men can lead to the expression of spiteful behaviors in which participants prefer to inflict damage to themselves at the expense of inflicting much greater damage to individuals of a competing group^15^.

Aggression and cooperation are multifactorial phenomena that are ubiquitous to human societies. Although aggression and cooperation seem to be opposite behaviors, they have several aspects in common. Both are generally employed to resolve conflicts over access to limited resources and social status. Therefore, they are important for success in mating^6,15–17^. Aggression can be considered a mechanism of intrasexual competition through peer domination^6,15,18,19^. In fact, the literature shows positive relationships between aggressiveness with dating^20^ and sexual activity^21^ in adolescents. In contrast, cooperation is a costly and honest signal of an individual’s ability to obtain a great amount of resources^22^, which allows him to share them with his peers for the benefit of the group. As a result, the cooperative individual is perceived as a valuable and resourceful ally, which in turn enhances the prestige and status of cooperative individuals^17,23^, increasing their mating opportunities. Accordingly, both behaviors are related to social status and reproductive success, although through different behavioral pathways^24^. In addition, in the specific context of intergroup conflict, cooperation and aggression are inevitable interdependent behaviors as men cooperate and form coalitions to outcompete other groups, or in other words, they cooperate to aggress.

Aggression and cooperation are affected by hormones, particularly by testosterone in males^9,25^. Testosterone (T) is a sex hormone associated with physical and psychological androgenization^26^. It has been well described that T activates male mating behavior in several species, including humans^27,28^. However, T is also of special interest in the study of aggression and cooperation because it influences the brain in situations strongly associated with the struggle for status^9,29^. The effect of T on behavior may be related to three ontogenetic moments: (1) circulating T levels, (2) prenatal T levels, and (3) pubertal T levels. Whereas circulating T levels exert an activation role on behavior, prenatal and pubertal T levels represent the developmental effects of testosterone, exerting an organizing role on the central nervous system, and through this, influencing lifelong behavior^30,31^. Given the difficulty of carrying out a cohort study, research on the relationship between developmental T levels and current behavior is centered on anthropometric traits, whose expression partly depends on developmental T levels. The length ratio of the second and fourth fingers (2D:4D) is commonly used as an indicator of prenatal T^32^, while facial masculinization^33,34^ and body muscularity^35^ are common T indicators of pubertal T.

Prenatal T levels are positively associated with aggressive dispositions, although the magnitude of the effect seems to be small^36,37^. Only two studies have investigated this relationship in the specific context of intergroup competition, and both had mixed results. McIntyre *et al*.^38^ used a war game in which participants played the role of the leader of a country in conflict with a neighbor over newly discovered diamond mines on disputed territory. The authors demonstrated that men with lower 2D:4D ratios (higher prenatal T) were more prone to make unprovoked attacks during the course of the game. In contrast, in a recent study, Isbell^39^ did not find any difference in 2D:4D according to decisions taken when subjects interact with teammates or rivals in the ultimatum game. Similarly, pubertal T levels, as assessed through cues like facial masculinity and muscularity through strength, have been shown to be positively associated with aggression^40,41^. In the specific context of intergroup competition, masculine faces are associated with wartime leadership^42^. Moreover, there is cross-cultural evidence supporting a positive association between body strength and the tendency to be more supportive of military action^43^, a proxy of the propensity to engage in serious intergroup conflict.

Prenatal T levels have also been shown to be positively associated with higher levels of cooperative behavior in a social dilemma and more generous offers in the dictator game^44^. However, the relationship between prenatal T levels and behavior was the reverse when aggressive cues were presented before participants began playing^44^, suggesting an important role of context in triggering cooperative or aggressive behavior. In general, the effect of 2D:4D on cooperative behavior is not consistent across studies. Studies have found that intermediate 2D:4D ratios are related to cooperative behavior in the prisoner’s dilemma and dictator games^45,46^. Millet and Dewitte^47^ investigated the effect of 2D:4D on contributions in a public good game (PGG) and found that individuals with lower 2D:4D ratios make minimal contributions to reaching the provision point (the threshold at which the public good is distributed among the players), considering equal contributions by all players, but neither less (selfish behavior) nor more (altruistic behavior). Less conclusive evidence has been reported concerning the role of pubertal T levels on cooperative behavior. First, studies have failed to show a relationship between facial masculinity and cooperation as assessed in the PGG^48^ or with the social value inventory^49^. Another study found that more muscular men were less oriented to egalitarianism^50^, that is, favoring equal benefits among group members. However, Stirrat & Perret^19^ found that facial masculinity is a positive predictor of cooperative behavior in PGG, but only in the context of intergroup competition. When participants play a PGG without the threat of another group, the relationship among traits is negative^19^. This suggests that the intergroup context is a key aspect in evaluating the effect of pubertal T on cooperation.

Another key prediction arising from The male warrior hypothesis is the effect of group formidability on the expression of both aggressive and cooperative behavior in the presence of an intergroup threat^8^. Individuals tend to assess group formidability from the overall body musculature of the outgroup^51^ and from the presence or absence of a successful outgroup leader^52^. Similar to the finding that more formidable individuals tend to behave more aggressively when facing a conflict^53^, it can be expected that individual behavior during intergroup conflict is influenced by group formidability^52^. In this sense, group formidability influences the likelihood of an escalating conflict in sports^54^.

**Figure 1:**
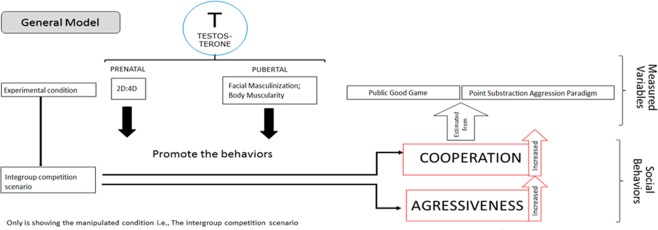
General research model.

The evidence reported in the paragraphs above indicates that developmental T, especially in the context of intergroup conflict, plays a key role in influencing behavioral changes related to cooperation and aggression, as predicted by the male warrior hypothesis. However, as far as we have been able to determine, there have been no studies that measure the three effects of developmental T on aggression and cooperation in intergroup conflict scenarios versus control conditions. This is important since T levels during developmental stages, especially during puberty, influence traits linked to physical strength, like skeletal muscle mass (SMM), which affect different behavioral manifestations^53,55^. Moreover, traits related to developmental T levels are thought to play an important role in intrasexual competition^56^ and, consequently, in intergroup conflict. In the present study, we used an experimental design to assess differences in aggression and cooperation based respectively on Cherek’s Point Subtraction Aggression Paradigm (PSAP)^57^ and a Public Good Game (PGG) under two conditions: intergroup conflict (21 groups of 6 individuals each), and a control condition in which the outgroup threat was removed (20 groups). We measured: (1) prenatal T levels (from 2D:4D), and (2) pubertal T levels (from facial width-to-height ratio, and body muscularity). Our predictions were as follow (Fig. 1):

- First, using a larger sample and a new population, we expect to replicate the previous results that indicate that intra-group cooperation and inter-group aggression are heightened in the context of intergroup conflict.
- Second, the use of aggression among human males is related to T-dependent physical traits. We expect this relationship to be positive in the contexts of both dyadic (one-to-one standard PSAP) and inter-group conflict. Accordingly, we expect a positive relationship between developmental T and aggressive behavior in the PSAP task under the control condition and in the context of intergroup competition.
- Third, similar to aggression, the use of cooperation is related to physical T-dependent traits, but in the opposite direction according to the context. More concretely, we expect a positive relationship between developmental T levels and cooperation in the intergroup conflict context, but a negative one in the control context, as previously reported^19^.
- Finally, we predict that individuals in more formidable groups, measured as the sum of group muscle mass or as the muscle mass of the most muscular individual in the group (the potential leader), show higher levels of aggression and cooperation than individuals in less formidable groups, but only in the intergroup competition context.

## Methods

### Participants

Over two years, 246 young men (mean = 22.21 years, standard desviation = 3.20) from public universities in the 5^th^ Region of Chile were recruited. Individuals were usually recruited as a group of 6 members who therefore knew each other. Four individuals were excluded because they did not complete the participation in all the games. We chose young adults because intrasexual competition and aggression are more intense in that period of life^58^. At the end of the experimental protocol, participants received $15,000 Chilean pesos each (around $23 USD) for participating. They received an additional payment of up to another $15,000 pesos according to their performance in the games. Thus, participants could receive a maximum of $30,000 pesos, and in fact, 90% of the participants received that amount. We decided to give a significant amount of money ($30,000 pesos represents 10% of the minimum monthly wage in Chile) to ensure interest and reliable participation.

### Ethics committee authorization and ensuring anonymity

The Institutional Bioethics Committee of the Universidad de Playa Ancha approved the research, including protocols and data treatment. All methods were performed in accordance with the relevant guidelines and regulations. Participants were asked to read and sign an informed consent form that detailed the procedure and the confidentiality steps. We used a standard coding process to preserve the anonymity of the participants^18,59^. All the participants signed the informed consent prior to their participation in the study.

### Group formation, context manipulation, and the data collection procedure

Each group of 6 participants was randomly assigned to one of two treatments, an experimental condition in which the intergroup competition scenario was presented, and a control condition in which no mention was made about an intergroup threat. There were 20 groups of each condition. More details of the conditions of the games are provided below. The games were conducted in the Laboratorio de Comportamiento Animal y Humano (www.labcah.cl) of the University of Playa Ancha, Chile. This laboratory has six experimental cabins with computers connected in a local network. The cabins are isolated from visual and audio stimuli. This ensures a high level of reliability in the performance of games, prevents talking among participants and favors their concentration. The data were taken in two sessions for each group, with one week between sessions. The first day, we applied a sociodemographic questionnaire (i.e. sexual orientation and age), conducted the Public Good Game, and took anthropometric measurements. The next week, participants performed the Point Subtraction Aggression Paradigm and received their payment. Groups were usually composed of individuals who were familiar with one another. However, in a few groups, some individuals did not know each other because they were friends of friends. We statistically controlled this heterogeneity of the group composition in terms of friendship.

### Anthropometric measurements

#### Indirect measurement of prenatal testosterone

Digit Ratio (2D:4D). Prenatal testosterone was inferred from measuring right-hand fingers based on earlier studies that indicate that prenatal T is most reliably estimated by this method^29,60^. We followed the protocol proposed by Manning^32^, and replicated by Muñoz-Reyes *et al*.^61^. We took two measurements of all the fingers and used the mean value from the two measurements. Measurements were obtained from the basal crease of the finger to the tip of the 2^nd^ and 4^th^ fingers. We used a high-precision digital caliper (±0.01 cm). The resulting variance (SD = 0.001) was similar to that obtained for this index in previous studies SD = 0.03 in^60,61^, which indicates a good level of precision.

#### Indirect measure of pubertal testosterone

Facial masculinity. Facial photographs in frontal view were taken of all participants with a digital SLR camera (Nikon D7000) under standardized conditions, in terms of light and head orientation, focal length (3 m), shutter speed (1/60 s) and aperture (f/5.6). Any facial adornments were removed, and participants were asked to look straight into the camera with a neutral expression.

Facial masculinity was based on the facial width-to-height ratio (FWHR), which was calculated using the vertical distance between the highest point of the upper lip and the nasion. We also measured facial width using the horizontal distance between the left and right zygion (i.e., bizygomatic width, the maximum horizontal distance between the right and left facial boundaries). Landmarks were located manually with TPS software. Finally, we compared our manual measurements with those obtained by the software FACE++, which locates and returns high precision facial landmarks. We automated the use of this software through an algorithm in MatLab created by the eighth author, which is connected to the Application Programming Interface of FACE++. There was a high degree of correlation between our fWHR measurements and those obtained from the MatLab algorithm (r = 0.82). The results were the same with either of the two FWHR measurements. Given the high degree of correlation between these methods, we preferred to use the manual measurements due to their proven utility in previous studies^62^.

Body Muscularity. We followed the protocol used by Muñoz-Reyes *et al*.^55^. We first measured the participants’ height in centimeters, barefoot, and with a manual stadiometer (SECA® 203). We then used the InBody® 370 body composition analyzer to estimate muscularity in kilograms. This device uses a tetrapolar 8-point tactile electrode to measure body composition by direct segmental multifrequency bioelectrical impedance analysis (DSM-BIA). This technique divides the body into five cylindrical parts before estimating impedance separately for each part, i.e., the four limbs and the trunk. The InBody® 370 applies three frequencies (5, 50, and 250 kHz) to measure impedance in the five segments. This methodology has been validated to assess body composition^63,64^. Bosy-Westphal *et al*.^63^ found that 97% of the variance in total SMM measured by magnetic resonance imaging was explained by SMM measured by DSM-BIA, whereas Ling *et al*.^64^ compared measurements of total lean mass of men measured using DSM-BIA with those obtained from dual energy X-ray absorptiometry, and found an intraclass correlation coefficient of 0.96. In addition, we collected data on the participants’ body mass index (BMI).

#### Behavioral measurements

For the baseline treatment, we use two experimental paradigms: The Point Subtraction Aggression (PSAP) and The Public Good Game (PGG). Whereas the PSAP paradigm has been used to elicit aggressive inclinations at the individual level in the context of dyadic one-against-one interaction, the PGG has been used to elicit cooperative dispositions in the context of a larger group social dilemma. These control conditions produce measurements of both cooperative and aggressive dispositions at the interpersonal level. The experimental conditions: the intergroup PSAP (IPSAP) and the intergroup PGG (IPGG) allows us to respectively explore how intergroup conflict modulates intergroup competition and intra-group cooperation. In all games where interaction was necessary with other men other than those in the group (i.e., dyadic and group conditions), participants were informed that they played with real people, although they were playing against a fictitious opponent (i.e., the software of the games).

Measurement of aggression. The Point Subtraction Aggression Paradigm (PSAP). First applied by Cherek in the 80 s, the PSAP is a highly reliable tool to estimate aggression, especially in men^65^. It consists of a computer game in which participants play against a fictitious opponent. Individuals are told that the objective of the game is to score the maximum points, which are exchanged for real money at the end of the game. The participant’s score is shown in a central monitor. Participants have three behavioral options that cannot be taken simultaneously:

- **Gaining points:** Participants gain 1 point by pressing button A 100 times. One point is equal to $1,000 Chilean pesos
- **Aggression:** Participants are informed that they can steal points from the other participant, but without gaining these points. Therefore, by pressing button B 10 times, they harm their adversary by subtracting one point, but without a concomitant increase in their own point total (i.e., stealing decreases the other player’s score without increasing one’s own). In addition, participants are told that their rivals get the points that are taken from them. To the extent that the only effect of stealing is to harm your opponent, stealing is consistent with the definition of aggression by Baron and Richardson^66^.
- **Protection:** Participants are told that their rivals can steal their points. Participants can avoid losing points by pressing button C 10 times, which protects them from points being subtracted in possible attacks during a fixed period of time.

We conducted a single 10-minute round. Participants under the control condition played the classic dyadic one-against-one version of the PSAP, while the participants under the experimental condition were told that they were part of a group competing with another group in a laboratory of another university located in the capital of the country. They were informed that each one was going to be paired with only one member of the competitor group, but that the winner would be the group that gained more points. The winning group would receive a bonus, equal to the points obtained by the losing group. This bonus would be split evenly between the members of the winning group. The losing group would only receive their individual points. Because the competitor group was fictitious, we always informed the participants that they had won the match, and gave them a bonus equal to 50% of the points obtained by themselves. To achieve more ecological validity and take into account the relevance of aggression in intergroup competition, but also for intragroup status, we followed the strategy used by Geniole *et al*.^65^. In this version, men are provoked intensely (i.e., participants lose 20 points per session). Aggression was calculated as the number of times button B was pressed as a percentage of the total number of times all the buttons were pressed. It is important to note that in our intergroup condition, conflict involves an outgroup threat with real potential consequences in terms of monetary payoffs, which the members of the group can collect by outcompeting the fictitious outgroup. We refer to this version of the PSAP as the Intergrupal PSAP (IPSAP).

Measurement of Cooperation. The public good game. As in any social dilemma, cooperating (any positive contribution) is a dominated strategy (i.e., a strategy that a selfish agent would never implement), but the absence of cooperation leads to an inefficient social outcome. Accordingly, the contributions of individuals can be used to assess their cooperative tendencies^67^. In the present research, we applied the protocol used by Van Vugt *et al*.^14^ and replicated by Stirrat & Perrett^19^ to measure changes in cooperation with the presence of intergroup conflict in the experimental condition. The public good game was played on computers using z-Tree software^68^.

Participants started the game with $5,000 Chilean pesos. They could decide how much to invest for the benefit of the group. They were told that they would receive a bonus of $11,000 pesos when total investment by the group exceeded $18,000 pesos, regardless of their individual contribution. However, no bonuses would be given if the group failed to contribute more than $18,000, and participants only gained the amount of money they decided not to share. Under experimental conditions, a group competed with another group for the bonus. As stated before, in the experimental procedure, the rival group was fictitious, although participants were informed that they played against a real group. The group of participants won the PGG if they contributed more than $18.000. Following previous research^14,19^, before playing the public good game, participants were provided with a complete description of the game and played a practice game. As part of a wider study, participants played two rounds of the game. The second round of the game was designed to study changes in contributions after winning or losing the first round. This was not included in this study as we were interested in the effect of the intergroup conflict in a one-shot cooperation. The difference between the design of Van Vugt *et al*. and ours is that, as in the IPSAP experimental condition, the outgroup threat involves real monetary consequences. In contrast, the strategy of Van Vugt *et al*. was that the introduction of intergroup conflict relies on priming inter-group competition. Specifically, PGG participants in the PGG from Southampton were told that the study was running simultaneously at 10 different universities in England.

To facilitate the interpretation of our results, let us discuss how the presence of inter-group conflict modifies the incentive structure that subjects face under the IPGG and the IPSAS experimental conditions. The only thing members of the group can do in the IPGG to outcompete other groups, and thus capture the winning prize, is increase intra-group cooperation. Alternatively, under the IPSAP scenario, besides continuing to gain points or defend against potential attacks (intra-group cooperation), stealing points from their opponents (inter-group aggression) also increases the likelihood of outcompeting their opponents.

### Data analyses

We conducted two t-tests with independent samples to test our first prediction by comparing mean rates of aggression and contribution according to whether individuals belonged to control or experimental groups. We also employed non-parametric Mann-Whitney U tests because rates of aggression and contribution in the PGG are non-normally distributed variables.

To test our second prediction, we fitted a general linear mixed model (GLMM) considering the following predictor variables: context (i.e., experimental or control), age, 2D:4D ratio, the facial width-to-height ratio, muscle mass, and body mass index. The rate of aggression in the PSAP was our outcome variable.

The third prediction was tested by a GLMM fitted to consider the following predictor variables: context (i.e., experimental or control), age, 2D:4D ratio, the facial width-to-height ratio, muscle mass, and body mass index. The outcome variable was the contribution. We expected an interaction between context and traits that denotes developmental T levels, and we took into account the interaction terms involving these variables.

To test our fourth prediction, we took the fitted model obtained for predictions 2 and 3 and tested whether the interaction of group formidability and context is significant. This assessed the potential effect of group formidability on individual expression of aggression and cooperation in a context of outgroup threat.

We used GLMMs to take into account the hierarchical nature of our data, in which we have individuals in groups and variables at the individual (e.g., muscle mass) and group levels (e.g., group formidability). We employed a step-up strategy to fit our models. In this procedure, all the predictor variables and the predicted interactions were compared individually with the null model. The variable that showed the best fit was introduced in the model. Next, we introduced the remaining variables one-by-one and compared their fit with the previous model (i.e., the reduced model). This procedure continued until no variables improved the reduced model. To compare nested models, we used the Akaike information criterion and the maximum likelihood estimation^69,70^. We considered SMM and BMI together on the one hand and the facial width-to-height ratio and BMI on the other when fitting the models to control for the effect of BMI on SMM and the facial width-to-height ratio. The model related to the aggressive response in the PSAP showed non-normal residual distributions. We transformed the variable rate of aggression as it was very right-skewed by calculating its square root. This transformation solved the problem of the non-normality of the residual. However, because the fitted models were the same as the original and transformed variables, we show the results with the original variable. We employed the statistical package HLM 7 to perform the GLMMs and the IBM SPSS 21 for the t-tests. The level of significance was set at alpha = 0.05.

## Results

### Differences in aggression and cooperation according to the competitive context

Table 1 shows the descriptive statistics of the variables used in the study according to the context. Table 2 shows Spearman’s correlation coefficients between these variables in the control and experimental contexts.

**Table 1:** Descriptive statistics and t-test for all predictors and outcome variables considered in the study.

	Control (N = 119)		Intergroup conflict (N = 123)			
	Mean	SD	Mean	SD	t-ratio	p-value
**Predictor**						
Age	22.00	3.03	22.37	3.37	−0.908	0.365
SMM	31.86	3.90	31.69	4.28	0.326	0.744
fWHR	2.19	0.18	2.20	0.20	−0.672	0.502
2D:4D	0.952	0.031	0.947	0.027	1.211	0.227
BMI	24.95	3.46	24.79	4.40	−0.270	0.788
Group formidability (Sum SMM)	192.02	10.13	190.14	11.63	−0.550	0.585
Group formidability (Max. SMM)	37.81	3.51	36.82	3.02	−0.970	0.338
**Outcome**						
Aggression	4.00	4.56	8.25	6.80	−5.689	<0.001
Cooperation	3327.34	1040.48	3655.66	1065.56	−2.424	0.016

**Table 2:** Spearman’s correlation coefficients between variables considered in this study.

	COOP.	AGGR.	SMM	FWHR	2D:4D	AGE	BMI
COOP.		r = −0.059 p = 0.517	r = 0.152 p = 0.093	r = −0.120 p = 0.187	r = −0.040 p = 0.662	r = −0.135 p = 0.135	r = −0.017 p = 0.853
AGGR.	r = 0.019 p = 0.837		r = 0.097 p = 0.287	r = 0.027 p = 0.764	r = 0.084 p = 0.354	r = −0.064 p = 0.482	r = −0.039 p = 0.666
SMM	r = −0.145 p = 0.115	r = 0.114 p = 0.216		r = 0.027 p = 0.764	r = 0.153 p = 0.091	r = 0.107 p = 0.237	**r = 0.564** **p < 0.001**
FWHR	r = 0.014 p = 0.878	r = −0.005 p = 0.959	**r = 0.208** **p = 0.023**		**r = 0.181** **p = 0.046**	r = −0.094 p = 0.301	**r = 0.197** **p = 0.029**
2D:4D	r = −0.168 p = 0.068	r = 0.066 p = 0.473	r = −0.107 p = 0.248	r = −0.016 p = 0.859		r = 0.172 p = 0.056	r = 0.151 p = 0.095
AGE	r = −0.008 p = 0.933	r = −0.065 p = 0.485	r = 0.069 p = 0.453	r = 0.089 p = 0.334	r = −0.037 p = 0.688		**r = 0.283** **p = 0.002**
BMI	r = −0.042 p = 0.651	r = 0.061 p = 0.513	**r = 0.565** **p < 0.001**	**r = 0.344** **p < 0.001**	r = −0.026 p = 0.782	**r = 0.226** **p = 0.013**	

Relationships between variables in the intergroup context (N = 123) are shown above the diagonal, and relationships between variables in the control context (N = 119) are shown below the diagonal.

Note: Coop.: Contribution in the PGG. Aggr.: Aggression in the PSAP.

The results of the t-tests showed differences in aggression between the two contexts in the PSAP (t = −5.722, df = 214.03, p < 0.001). Individuals in the intergroup competitive context showed higher rates of aggression (mean = 0.083, SD = 0.070) than individuals in the control condition (mean = 0.040, SD = 0.046). The results of the non-parametric test point to the same pattern (Mann-Whitney U test: U = 4335.50, n_1_ = 119, n_2_ = 123, p < 0.001). As expected, the rate of aggression in the PSAP correlated negatively with profits (Pearson correlation coefficient: r = −0.549, N = 242, p < 0.001) and consequently, individual profits in the experimental condition (mean = 22.36, SD = 5.92) were lower than in the control condition (mean = 24.80, SD = 5.78) (t = 3.244, df = 240, p = 0.001).

The results of the t-tests showed differences in cooperation during the PGG round according to the context (t-test for independent samples: t = −2.424, df = 240, p = 0.016). Individuals facing an intergroup competitive context contribute on average $328.3 Chilean pesos more (mean = 3655.66, SD = 1065.56) than individuals in the control condition without the outgroup threat (mean = 3327.34, SD = 1040.48). The results of the non-parametric test point to the same pattern (Mann-Whitney U test: U = 6153.50, n_1_ = 119, n_2_ = 123, p = 0.030). To further explore the effect of context in cooperation, we analyzed cooperation between participants according whether they invested at least the minimum amount to reach the threshold considering an equal contribution. We found that the effect of context on cooperation was non-linear. Whereas the percentage of individuals that decided to invest $3,000 pesos or more did not differ between conditions (Х^2^ = 0.608, df = 1, p = 0.435), the individuals that invested $3,000 pesos or more in the control condition contributed less (mean = 3719.72, SD = 673.70) than those in the experimental condition (mean = 4002.51, SD = 762.63) (t = −2.742, df = 194, p = 0.007), but there were no differences among individuals that invested less than 3,000 (control condition: mean = 1852.00, SD = 832.85; experimental condition: mean = 1970.95, SD = 628.66) (t = −0.538, df = 44, p = 0.593).

We did not find differences between the contexts for any of the predictor variables (see Table 1).

### Predictors of aggression in an intergroup conflict scenario

**Figure 2:**
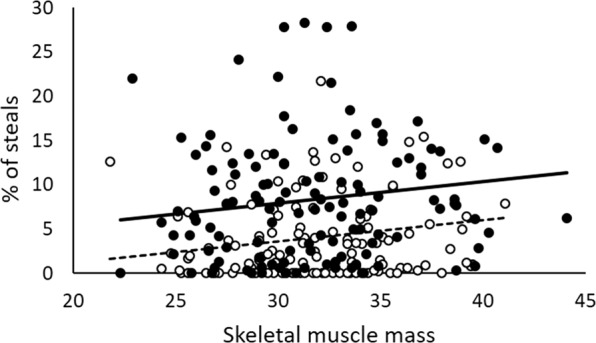
Relationship between skeletal muscle mass and the % of stolen points in the PSAP according to the context. Dots represent observed values in the control (empty dots) and intergroup conflict (full dots) contexts. Lines represent expected values across the observed range for control (slashed line) and intergroup (continuous line) contexts. Expected values are evaluated at the mean value of BMI (24.72).

Table 3 shows the fitted model considering prenatal and pubertal markers of T levels according to the context. In addition, we considered age and BMI as control covariables. We found that muscular mass was a positive predictor of aggression in both contexts (B = 0.238, t = 2.294, p = 0.023; see Fig. 2). However, neither the facial width-to-height ratio nor the index 2D:4D were significant predictors of aggression in the PSAP, regardless of the context. We found a main effect of the context (B = −4.299, t = −4.282, p < 0.001). Individuals in the intergroup conflict context were 2.08 times more aggressive than individuals in the control condition when muscular mass and BMI were evaluated in their means (control context: mean = 3.971, SD = 0.717; intergroup context: mean = 8.270, SD = 0.702). These results further support the previous finding of the effect of context on aggressive behavior.

**Table 3:** Fitted model for the relationship between T developmental traits and aggression.

Parameter	Estimate	Std. Error	df	t	Wald Z	p
***Fixed effect***						
Intercept	6.028	2.978	240.3	2.040		0.042
Context = 0	−4.299	1.004	41.3	−4.282		<0.001
SMM	0.240	0.104	236.3	2.294		0.023
BMI	−0.215	0.107	236.9	−2.004		0.046
***Covariance parameters***						
Intergroup	6.260	2.502	39		91.421	<0.001
Residual	27.314	5.226				

Estimates of fixed effects and covariance parameters.

Context: [0 = control condition, 1 = intergroup conflict condition].

SMM: Skeletal muscles mass.

BMI: Body mass index.

### Predictors of cooperation in an intergroup scenario

**Figure 3:**
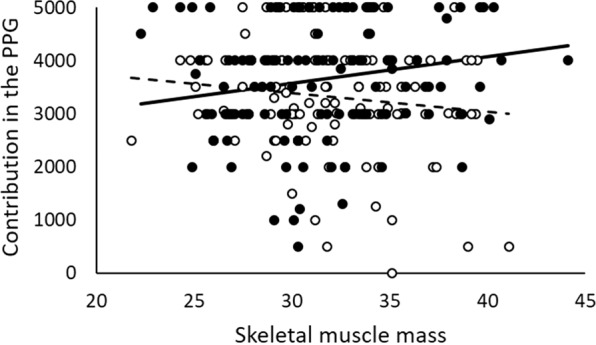
Relationship between skeletal muscle mass and the contribution in the PGG according to the context. Dots represent observed values in the control (empty dots) and intergroup conflict (full dots) contexts. Lines represent expected values across the observed range for control (slashed line) and intergroup (continuous line) contexts. Expected values are evaluated at the mean value of BMI (24.72).

Table 4 shows the fitted model considering prenatal and pubertal markers of T levels and their predicted interactions according to context. We also considered age and BMI as control covariables. We found an interaction between context and muscle mass. Muscle mass was negatively related to contributions in the PGG in the control context (B = −85.633, t = −2.624, p = 0.009; see Fig. 3), but positively related in the intergroup conflict context (B = 50.818, t = 2.097, p = 0.037), which partially supports our expectation about the relationship between developmental T levels, context and contribution. Nevertheless, the other traits signaling developmental T levels, the facial width-to-height ratio and the 2D:4D ratio, were not significant predictors of cooperation in either context.

**Table 4:** Fitted model for the relationship between T developmental traits and cooperation. Estimates of fixed effects and covariance parameters.

Parameter	Estimate	Std. Error	df	t	Wald Z	p
**Fixed effect**						
Intercept	2859.83	712.74	242.0	2.012		<0.001
Context = 0	2386.89	1045.63	242.0	2.283		0.023
SMM	50.82	24.23	242.0	2.097		0.037
BMI	−32.87	19.90	242.0	−1.652		0.100
Context = 0*SMM	−85.63	32.63	242.0	−2.624		0.009
**Covariance parameters**						
Intergroup	2703.06	51.99	39		35.675	>0.500
Residual	1078787.15	1038.65				

Context: [0 = control condition, 1 = intergroup conflict condition].

SMM: Skeletal muscles mass.

BMI: Body mass index.

### Are aggression and cooperation linked to group formidability?

Regarding aggression, we found that the sum of SMM in the group as a measure of group formidability was not related to the number of points subtracted in the PSAP, either as a main effect (B = −0.049, t = −1.002, p = 0.321) or in interaction with the context (B = 0.115, t = 1.239, p = 0.222). Similar results were found using the maximum individual SMM of the group as a measure of group formidability regarding main effects (B = −0.121, t = −0.750, p = 0.457) and the interaction with the context (B = 0.470, t = 1.514, p = 0.137).

Regarding cooperation, we found that the sum of SMM in the group was not related to contributions in the PGG, either as a main effect (B = 1.865, t = 0.270, p = 0.787) or in interaction with the context (B = −14.448, t = −1.041, p = 0.299). Similar results were found using the maximum individual SMM of the group as a measure of group formidability regarding main effects (B = −19.077, t = −0.864, p = 0.388) and the interaction with the context (B = −69.333, t = −1.564, p = 0.119).

## Discussion

In this paper, we tested several predictions derived from the male warrior hypothesis^8,14^. First, we replicated previous results^14,15,19,71^ about the importance of the intergroup conflict scenario in promoting cooperation within group members and aggression toward outgroup members. We then tested specific predictions about the hormonal underpinnings of male cooperation and aggression during intergroup conflict, concretely the role of an indirect measure of developmental T levels in both behaviors and in a context of intergroup conflict versus a control context without an outgroup threat. In this case, we found only partial support for our predictions since only muscle mass, an indirect marker of pubertal T levels, seems to be associated with aggression and cooperation in the predicted direction when the context is taken into account. Finally, we did not find evidence that the formidability of the group affected individual rates of aggression or cooperation, controlling for individual characteristics.

The male warrior hypothesis is founded on the importance of intergroup conflict for the reproductive success of individuals, especially men. This framework argues that men have physical and psychological traits selected in the context of intergroup competition. Several investigations have shown that men, in fact, tend to show ingroup favoritism and outgroup hostility, a phenomenon known as “parochial altruism”^72–74^. In this study, we replicated this finding showing that on average individuals were more cooperative in a public good game, that is, they contribute more to the common pool when they competed against another group in order to first reach a threshold, than when they played the game in order to reach the same threshold but without the threat of an outgroup. Further, although most individuals contributed 3,000 Chilean pesos or more, which was the minimum amount to reach the threshold considering an equal contribution, we found that among individuals that contributed 3,000 pesos or more, those in the experimental context behaved more altruistically, that is, they contributed more than those in the control condition. The contributions of individuals that did not invest at least 3,000 pesos were not different between the two conditions. In other words, individuals in the experimental context decided to invest far more than the minimum to reach the threshold under an equal contribution. If one assumes that there is an implicit norm under this scenario, namely to contribute 3,000 pesos, the difference between both treatments is driven by the supererogatory behavior of some agents whose extra contribution could be understood as a status-seeking strategy. Moreover, individuals behaved more aggressively during the PSAP when they were collectively competing against another group (experimental condition) than when they were competing individually against an individual from an outgroup. Then, aggression was heightened by intergroup conflict even if this aggression was in some sense spiteful, as it was costly for the aggressor and the receiver. In fact, rates of aggression correlated negatively with profits in our study, and the average benefits of individuals in the intergroup conflict context were lower as a consequence. Our results support previous findings about the importance of intergroup competition in cooperation with members of the group and aggression against the members of a competing group.

We were also interested in the hormonal underpinnings of this phenomenon. Testosterone is an androgenic hormone that, among other functions, has been proposed to be a key factor in calibrating cooperative and aggressive responses in different contexts^9^. More concretely, the indirect measures of developmental T levels are expected to be associated with aggressive responses in general and intergroup conflict scenarios^37,38,41,42^. In this study, we tested the indirect effect of developmental T levels with rates of aggression in a context of intergroup conflict and in a control context. First, we found some support for the claim that an indirect measure of developmental T levels is important in determining levels of aggression. Concretely, we found that body muscularity is a positive predictor of rates of aggression in the intergroup conflict scenario, as well as in the control scenario. However, we did not find any effect of the fWHR or 2D:4D. The fWHR has been related to aggression, both self-reported and as measured by the PSAP^30^, although the relationship between the fWHR and aggression may be moderated by social status^75^. However, a recent study that evaluated the fWHR and bicep circumference found that bicep circumference was a significant predictor of aggression in the PSAP, while the fWHR was not^76^. Our results are in accordance with those of the latter study in suggesting that body muscularity is the key factor related to aggression and that the fWHR is a correlate of potential physical threat^76^. Evidence of the relationship between 2D:4D and aggression comes mainly from self-reports of aggressive behavior rather than behavioral measurements obtained in a laboratory paradigm, and their effects are small^37^. In our study, 2D:4D was not a significant predictor of aggressive response in the PSAP in either condition. 2D:4D is probably a reliable indicator of psychological predisposition to aggression, but the real physical power of individuals limits this predisposition under realistic conditions. Using a war game, McIntyre *et al*.^38^ found more strategic than direct use of aggression. Another explanation of this null result can be found in the proposal of Manning *et al*.^29^ of an indirect effect of 2D:4D on competitive/aggressive behavior, in which 2D:4D predicts spikes in circulating T, which is a promotor of aggressive behavior through increases in T. Future studies should include measuring circulating T and other forms of aggression to discard or demonstrate a role of 2D:4D in aggressive behavior in the scenario of intergroup conflict.

Regarding cooperative behavior, we found that muscle mass was an important variable in determining individual levels of contribution in the Public Good Game. The effect was moderated by context, that is, more muscular men behaved more cooperatively in the intergroup conflict condition and less cooperatively in the control condition. This supports the prediction that the indirect measure of developmental T levels enhances ingroup cooperation when facing an outgroup threat. These results are similar to those of Stirrat & Perret^19^, who showed that the fWHR is related to contributing more or less in a Public Good Game, depending on the context (between groups versus within groups). However, we tested both fWHR and muscle mass and only found a significant effect of muscle mass in cooperation. This result adds further evidence about the importance of the intergroup context in moderating the relationship between the indirect measure of pubertal T levels and cooperation in men and suggests that muscle mass plays a more prominent role in cooperation during intergroup conflict. Less muscular men behaved more cooperatively under our control condition. A possible explanation is that our indirect measure of pubertal T levels, muscle mass, enhances anti-social behavior when there is no outgroup threat. The latter probably occurs because physical power is a reliable cue for fighting ability^18,77,78^ that serve to subdue ingroup rivals and acquire social status and benefits through the use of non-cooperative displays (for instance, the use of anger in^53^. Given that most individuals contributed at least 3,000 pesos in the Public Good Game, that is, most individuals cooperated in the game, we suggest the control condition can be understood as a scenario based on the balance of cost-benefits of obtaining social status through prestige. In the context without outgroup threat, individuals with traits denoting lower indirect measure of pubertal T levels have the opportunity to gain status through prestige in a context in which competitive traits are less important^24^. However, when cooperation is triggered by intergroup competition, individuals with traits denoting indirect measure of pubertal T can be expected to cooperate more^14,19^. We speculate that this is a reliable way to maintain previously acquired ingroup status, and to maintain group structure. According to prenatal T, we did not find any relationship between 2D:4D and cooperation. Although previous studies have reported inconsistent results about the relationship between 2D:4D and cooperation^44–46,79,80^, it is plausible that indirect measure of prenatal T has a more indirect role in the expression of cooperation in the intergroup conflict scenario, as with aggression. In a study with female subjects^81^, inoculating testosterone only increased cooperation among participants with a lower 2D:4D ratio.

We did not find evidence that individuals adjust their behavior according to the formidability of the group. This is a key prediction of the male warrior hypothesis^8^, which, to our knowledge had not been tested until now. There are several possible explanations for the absence of any indication of the influence of group composition. Our expectation that individuals would adjust their behavior in the same way according to the composition of the group may have been too simplistic. It is possible that the effect of group formidability only manifests itself with a few individuals who act as leaders, while weaker individuals rely on the strength of the leaders. Another explanation is that because members of one group are not able to assess the formidability of the outgroup, they calibrate their behavior based on their day-to-day experience about the formidability of the group, which may vary among individuals in the same group and among groups. In any case, previous studies suggest that individuals can assess the formidability of other groups. Therefore, future studies should focus on determining if this ability translates into calibrating individual behavior according to group composition compared with the outgroup.

Our study has several limitations that have to be considered in appraising the scope of our results. First, we have not measured the degree of friendship among the participants. However, our statistical design allowed us to control for variability in aggression and cooperation between groups. In any case, we are beginning to test this variable for a new project, and although it is clear that an interaction scenario affects the behavior of participants, it would be interesting to test the possible effect of the previous history of the relationship on strategic behavior in the context of both aggression and cooperation. In addition, we have not considered the inclusion of psychological variables that could moderate the expression of the tested behavior, such as self-perceived social status, general aggression, and social value orientation. Finally, although we have demonstrated that outgroup aggression and ingroup cooperation are exacerbated among men engaged in intergroup contests, we cannot deny the possibility this effect is also present among women. In the future, we expect to study this issue among women.

To conclude, our results from applying an experimental design under controlled conditions support one of the main predictions of the male warrior hypothesis, that aggression and cooperation are heightened in groups of men in the context of intergroup conflict. Our analysis of the effects of developmental testosterone on aggression and cooperation further supports the notion that body muscularity is an important trait that influences the intensity of aggressive responses under provocation, as measured in the PSAP. Notably, the relationship between body muscularity and aggression is not dependent on the context (intergroup conflict versus control). In our study, the context of intergroup aggression increased the rates of aggression independent of testosterone. In other words, men seem to increase aggression regardless of their body muscularity. Our results indicate that context influences and increases cooperative behavior in men. Muscularity affects cooperativeness, more muscular men being more cooperative than less muscular counterparts in the intergroup scenario, but with the reverse effect in the control situation (only with the intragroup scenario).

Future studies are needed that include circulating T andanalysis of this effect in interaction with anthropometric indicators of developmental T levels. More complex experimental designs are needed to include the assessment of the rival group’s formidability. The mechanisms (if they exist) are more likely to be noted if a visual comparison is made before the competition.

## Data Availability

The data set generated and employed in this study is available from the corresponding author upon request.
